# The Mediation Role of an Individual’s and Couple’s Psychological Factors, Including Parenting in the Prediction of Relational and Marital Satisfaction

**DOI:** 10.3390/ijerph191711011

**Published:** 2022-09-02

**Authors:** Roxana Chiș, Sonia Ignat, Dana Rad, Irina Macsinga

**Affiliations:** 1 Faculty of Educational Sciences Psychology and Social Sciences, Aurel Vlaicu University of Arad, 310032 Arad, Romania; 2 Department of Psychology, West University of Timisoara, 300223 Timisoara, Romania

**Keywords:** relationship satisfaction, marital satisfaction, marital adjustment, personality, sustainability, parenting correlates, mediation

## Abstract

The goal of this study was to widen the scope of the social component of family sustainability. The fundamental goal of this non-experimental, quantitative study was to look at the links between parenting correlates, personality characteristics, marital satisfaction, and well-being in couples, as well as to construct explanatory prediction models for relational and marital satisfaction. The study focused on the effects of personality traits, parental self-efficacy, and attachment to marital and relational satisfaction. The test instruments applied were: the Experiences in Close Relationships-Revised, Marital Adjustment Test, Couple Satisfaction Index, Relationship Satisfaction Scale, Family Distress Index, Generalized Self-Efficacy Scale (adapted to the role of a parent), Mowen’s Personality Scale, and demographic data. A total of 238 Romanians answered the online survey, recruited based on a convenience sampling method. Participants in this research were individuals who were both married and had at least one child. The main findings show that agreeableness, openness, conscientiousness, anxious and avoidant attachment, and marital adjustment predict the satisfaction in the relationship, and openness partially mediates the association between marital adjustment and relationship satisfaction. Parental self-efficacy appears to influence the association between relationship satisfaction and marital satisfaction. An increased parental self-efficacy score predicts an increased relationship satisfaction and marital satisfaction. The higher the parental self-efficacy, the higher the satisfaction in the relationship, which leads to an increase in satisfaction in the couple. These results show that marital adjustment and adaptation are related to relational satisfaction, and these relationship are partially mediated by an individual’s openness. Despite some limitations, the current study significantly contributes to couples therapies and interventions in terms of physical and mental health, and the study provides insight into the experiences and perspectives of married individuals with children in Romania.

## 1. Introduction

The goal of this study was to widen the scope of the social component of family sustainability by extending the knowledge developed in other nations to Romania and testing the generalizability of previous research. Parents have as their main responsibility the upbringing of their children [1,2]. The main objective of parents, considering their position as adults in contrast to the child that later grows into an adolescent, is none other than to foster the psychosocial development of their children so that they become healthy adults and responsible members of their society [3,4]. The child achieving a good psychosocial development is not just the implication that they have a good social adjustment without developing psychological maladjustments [5], aggressive behaviors [6], antisocial tendencies [7] or, already in adolescents, initiation in drug use [8].

Moreover, especially in the context of the digital society, good child development also includes indicators of adjustment based on health and well-being [9,10]. For example, parents should help children to develop a good self-concept [11], self-esteem [12], prosocial behaviors [13], empathy [14], maturity [15], and school performance [16]. The socializing task itself can be an important source of attrition for couple relationships because of high parental stress [17], but the task can also bring spouses closer together [18].

Therefore, the socializing task is arduous and complex for parents and lasts, in most societies, for almost two decades [19,20]. However, parents are not merely educators whose union is limited to the transmission of social and cultural values [21,22]. Parents form a couple united by love and commitment [23,24]. While many parents separate, many others also remain together during the socialization of their children, with differences between parents with a good relationship [25,26] and others with a poor and deteriorating couple’s relationship [27,28].

For years, one of the most studied topics in the family literature has been the contribution of parents to the psychosocial development of children (i.e., parental socialization), which has been studied through the two-dimensional theoretical model with two theoretically orthogonal dimensions [19,29]. On the one hand, the model includes the affect dimension [17,30] and the severity dimension on the other [31,32]. Generally, parental socialization is one of the most important topics in the literature concerning family [2,33]. However, less is known about parental adjustment and how the parents’ relationship as a couple affects them as individuals, as well as their parental competence [24,25,27]. The parental adjustment and relationship between the spouses in turn affects the socialization process itself and thus child development. The development of children is important, but so is the parental adjustment, which in turn depends on many factors (although the couple’s relationship may be a key factor). Among the limited evidence, some of the evidence suggests that the better the relationship between spouses, the better the adjustment and parenting ability of fathers [24,26], although this is not definitive since it has also suggested that the relationship between spouses is independent of both their adjustment and their parenting.

This quantitative study examined the possible links between personality characteristics and parenting correlates, as well as marriage and relationship satisfaction. Several fundamental theoretical factors have been identified by academics as strong predictors of relationship happiness or unhappiness. Personality qualities, emotional communication, gender orientation, problem-solving, aggressiveness, parental self-efficacy, time spent together, financial conflict, duration of marriage, and the presence of children are all predictors [34,35]. If these variables have a positive or negative influence on relationship satisfaction when independently considered, the interaction between them adds an explanatory layer to the variance in satisfaction.

Several fundamental theoretical factors have been identified by academics as strong predictors of relationship happiness or unhappiness [36,37]. For many individuals, the couple partnership is the most significant and long-lasting social contact in their lives. Intimate relationships that are stable have been associated with decreased rates of depression, greater levels of life satisfaction, and overall happiness [36,38].

High degrees of happiness in romantic relationships have been linked to significantly positive results, which include a lower degree of relationship dissolution and higher relationship stability [39], as well as assisting mental health and establishing a greater sense of well-being [40].

The personality traits of romantic partners can contribute to the level of their relationship satisfaction. Marital adjustment is a broad term that refers to how a married pair functions together. Some researchers have applied the notion to define the meaning of marital adjustment and to evaluate the marital relationship’s quality [41]. A subjective assessment of a married couple’s relationship by their partners can be defined as the quality of their marriage [42].

According to Heyman [43], marital satisfaction is a multifaceted notion that encompasses a variety of qualities regarding the marriage, such as adjustment, happiness, integrity, and commitment [44]. Various components of the marital connection combine to produce the multidimensional idea of marital satisfaction [45].

Particularly in the early years of marriage, when marital satisfaction is unstable and the marital relationship is at risk, marital contentment is a mental condition that demands that the couples make continual efforts to actualize it [46]. When a couple’s marriage is in line with their expectations, they feel fulfilled as a pair [47].

The subjective assessment of a partnership is known as relationship satisfaction. Relationship satisfaction is a subjective emotion and viewpoint, not a characteristic of a relationship. As a result, spouses from the same marriage may have differing levels of relationship satisfaction [48].

The attitude a person has toward their own marriage may be referred to as marital satisfaction. The study of marital satisfaction has significant ramifications for relationship researchers as well as practical ramifications for the married individuals, as well as for the professionals who deal with them [49]. Parenthood has had a higher detrimental impact on marital satisfaction in recent years, with younger birth cohorts, and high socioeconomic categories. The evidence indicates that role conflicts and restrictions of freedom cause marital satisfaction to decline following the birth of a child [50]. The transition to motherhood is another event that frequently poses a barrier to relationship satisfaction [51,52]. Numerous studies have indicated that following the birth of the first child, relationship satisfaction significantly decreases [50,53,54].

Marital adjustment is defined here by using concepts that have been circulated in the literature before, such as: marital satisfaction, the degree of affection of the partners in the couple, and the level of cohesion [41]. From this perspective, marital adjustment is a dynamic process that can be placed on an evaluative continuum, from poorly adapted couples to very well-adapted couples [55]. It can have an impact on the quality of a marriage, but it should not be considered one of the concept’s elements, as adjustment or adaptation is an aspect that refers rather to the relationship that marital partners have rather than the feelings that each person experiences in the relationship. Adaptation indicators (e.g., communication, conflicts between partners, carrying out joint activities, etc.) may relate to the feelings experienced by the two spouses in different ways [55].

Literature where the focus has been on marital adjustment cannot be completely separated from the literature that has focused on the concepts of marital relationship quality or marital happiness. This is because some methods that are designed to assess the marital relationship quality mix items that evaluate adjustment with items that evaluate the assessments that marital partners make in relation to various aspects of their relationship. The Marital Adjustment Test [56] is an example of such an instrument, and it is a scale that has been used in the present study. Personality aspects that have shown to have significant relationships with certain indicators of marital quality of life include: impulse control, interpersonal conflict resolution styles, neuroticism, extraversion, orientation toward conventional behaviors, attitudes, self-esteem and interpersonal skills, and adult attachment styles [57].

Certain patterns of personality traits present in the marital partners may predispose them to distorted interpretations of events that they experience in the marital relationship or to some, create exacerbated reactions to negative events, which lead to difficulties in marital dyad relationships [42]. Several studies have shown that personality traits that have been described by the Big Five Model significantly predict the marital adjustment [42]. In a longitudinal study, it was discovered that, when compared with the partners’ educational level, history of past divorces, or age of the marital partners, the personality factors that were present were stronger predictors of marital instability, which was assessed by the researchers four years later [58].

Significant associations have been demonstrated between personality traits and the level of marital adjustment [58]. Therefore, high levels of psychoticism tend to be inversely related to the marital adjustment level, while high levels of internality and agreeableness tend to be positively associated with marital adjustment. Studies have shown that in addition to these specific traits, the clarity of emotional expressiveness and tendency to understand the perspective of the partner tend to positively correlate with the marital adjustment, while ambivalence in the field of expressiveness tends to be a negative predictor of marital adjustment [58]. The results regarding the role that extraversion plays in predicting marital adjustment are intertwined. Several studies have discovered a tie between marital adjustment and extraversion; some have identified a negative relationship, while other research studies have shown no evidence of a link between these two variables.

Attachment is a key component to consider when selecting a mate, as well as in the functioning of the relationship [57,59]. Secure attachment partners were content with their relationship, partner, and lifestyle [59,60]. A disorganized attachment style adds a new component to the adult attachment system, the partners’ fear, which is a more basic and widespread type of fear, known as “fear without a solution” [61]. Thus, partners with a disorganized style of attachment show justified or unjustified fear about their partner and their partner’s actions, while also declaring that this fear leads to quarrels, conflicts and dissatisfaction in the couple’s relationship [61].

Self-efficacy is a person’s belief in his or her own ability to successfully carry out a certain task or activity. Self-efficacy can highlight details regarding how a person might behave, how they may try to perform a task, how much effort they put into performing the task, and how long they persist in the face of challenges [62,63]. Bandura (1997) created the phrase “self-efficacy” to further develop cognitive social theory, which explains a person’s performance in specific tasks. According to this author, individuals rely on four sources: personal interpretations of their own performance and abilities, observing how others perform a task, their reaction to social persuasion, and their individual physiological and emotional state [64]. The four sources were incorporated into Gist and Mitchell’s (1992) model of the relationships between performance and self-efficacy, which was based on social cognitive theory [65]. Parental self-efficacy refers to a parent’s or caregiver’s belief in their own abilities to successfully raise children [66].

Parental responsiveness and parental demandingness are two crucial aspects of parenting that are captured by the parenting style [29]. Parental responsiveness, also known as parental warmth or supportiveness, is the degree to which parents consciously promote uniqueness, self-control, and self-assertion by being aware of, accommodating of, and obedient to children’s unique needs and expectations [67]. Parental demandingness, also known as behavioral control, describes the demands that parents place on their kids to become a part of the family as a whole, including their expectations for maturity, efforts to monitor them, and readiness to discipline any disobedient kids [67]. A typology of four parenting styles: indulgent, authoritarian, authoritative, and uninvolved is produced by classifying the parents based on how demanding and responsive they are as parents [29]. Each of these parenting approaches exhibits a certain ratio of response to demands, as well as various naturally occurring patterns of parental attitudes, practices, and behaviors [68]. According to Desjardins, the parenting style can predict a child’s wellbeing in the areas of social skills, academic achievement, psychosocial development, and problem behavior [69]. Children and teenagers with authoritative parents estimate their own social and instrumental competence to be higher than those that lack authoritative parents, according to objective measurements [70]. Children and young people with uninvolved parents perform the worst in all aspects. Parental responsiveness predicts social competence and psychological adjustment in general, but parental demandingness predicts instrumental competence and behavioral control (academic performance and deviance). As a result, the most appropriate approach for our study was to design a non-experimental study.

### Objectives of the Study

The fundamental goal of this non-experimental, quantitative study was to examine the links between parenting correlates, personality characteristics, marital satisfaction, and well-being in couples, as well as to construct explanatory prediction models for the two dependent variables. This study focused on the effects of personality traits, marriage duration, level of education, parental self-efficacy, attachment, and level of education on marital and relationship satisfaction; it was conducted with marital satisfaction and relationship satisfaction being regarded both as independent variables and dependent variables.

Previous studies have demonstrated that personality traits and parenting factors play an essential role in successful relationships [34,71,72]. As a result, we desired to provide more information regarding the links between these two variables through our research of couples’ personality traits, parenting correlates, and marital satisfaction and well-being. There has been little research on the influence or link that personality characteristics and parenting correlates have on marital satisfaction and well-being in a marriage. In the determining of the core components that promote relationship happiness, several of the findings were found to be inconsistent. While some researchers discovered that comparable personality qualities resulted in relationship satisfaction [73], others discovered that complimentary personality traits benefited partnerships, and couples with similar personality traits reported lower relationship satisfaction over time [71,72].

In order to fulfill the aforementioned goals, the following working hypotheses were formulated:

**H1.**  *Relationship satisfaction is significantly predicted by attachment styles measured with ECCR-R and marital adjustment, assessed with the MAT scale, controlling for the personality dimensions, agreeableness, openness, and conscientiousness*.

**H2.**  *Openness to experience partially mediates the relationship between marital adjustment and relationship satisfaction*.

**H3.**  *Marital (couple) satisfaction is significantly predicted by marital adjustment and anxious and avoidant attachment, controlling for the personality traits of agreeableness, openness, and conscientiousness*.

**H4.**  *The association between marital satisfaction and relationship satisfaction is partially mediated by parental self-efficacy*.

## 2. Materials and Methods

### 2.1. Participants

This study’s target population consisted of Romanian adults, recruited based on a convenience sampling method. The participants in this research were not dyads, but were all married individuals and also parents.

Convenience sampling, often referred to as incidental sampling or grab sampling, is a non-probability sampling technique in which researchers select their sample only by convenience. In convenience sampling, the only selection criterion is the ease of recruiting the participant; there is minimal judgment or conjecture involved. Because the sample is not chosen at random but rather by the researchers, not every member of the population has an equal chance of taking part in the study. Costs, regional dispersion, or the ease of gathering data may be obstacles for participation. Convenience sampling techniques include inviting individuals to take part in the study, gathering information from local sites, mailing out surveys, and posting links on social media. Because convenience sampling has few criteria to follow and allows for the quick generation of large samples, it was selected as the preferred selection type in this study. The second reason for choosing convenience sampling was that it would gather answers from populations that were easy to reach. The third reason for choosing the convenience sampling method was to gather preliminary data and investigate hypotheses that might be tested in subsequent studies, since the data are easily accessible. Regarding the drawbacks of convenience sampling, instances of biases that may emerge from this sort of sampling are positivity bias, selection bias, and sampling bias.

The online questionnaire was distributed via social media platforms and completed by 238 individuals, of which 76 were male (31.9%) and 162 were female (68.1%). We intended to request individual answers rather than responses from the couples, in light of the COVID epidemic. The age categories were between the ages of 18 and 25 (3.8%), 26 and 35 (26.5%), 36 and 45 (34%), 46 and 55 (34%), and over the age of 55 years (4%).

In terms of education, there were 131 participants with a secondary education (55%) and 107 participants with a higher level of education (45%). In terms of the duration of marriage, there were participants that had a marriage duration from 5 to 8 years (N = 78, 67.2%), and others that had a marriage duration from 9 to 10 years (N = 160, 32.8%).

### 2.2. Measures

The ECR-R—Experiences in Close Relationships-Revised [74]—questionnaire consists of 36 distinct items that measure the style of attachment in adults. The ECR-R measures the attachment of individuals on two attachment subscales: avoidant attachment and anxious attachment [74]. It comprises 18 Likert-type items that evaluate anxious attachment and 18 items that evaluate avoidant attachment. Items were rated on a scale between 0 (total disagreement) and 6 points (total agreement). Participants were instructed to consider the overall romantic/love connection experiences, including from both previous and current relationship experiences, and to respond based on these experiences. An example of an item is, “I do not often worry about being abandoned”. The scale has a very good reliability in each dimension, with a Cronbach alpha of 0.94 on the anxious attachment side and 0.95 in the avoidant attachment side.

The MAT, or Marital Adjustment Test [56], is a 15-item scale that measures the marital adjustment. It was originally used to differentiate well-adapted couples in marital relationships from couples experiencing difficulties. According to reports, the MAT’s reliability falls between Cronbach alphas of 0.72 and 0.83 [75,76,77]. Other subsequent research has demonstrated a substantial difference between the satisfied and unsatisfied marriage sample for both men and women, demonstrating the validity of the MAT scale [78,79,80]. The MAT examines a variety of response scales, including ordinal and Likert scales. Item 1 is rated on a seven-point Likert scale (ranging from “Very Unhappy” to “Perfectly Happy”); items 2–9 are evaluated on a standard six-point scale (from “always agree” to “always disagree”). Item 10 requires the respondents to select one option out of three choices. Item 11 makes use of a four-point ordinal scale (ranging from “All” to “None”). Item 12 invites respondents to select one of two choices concerning the connection between themselves and their spouse. Item 13 makes use of a four-point ordinal scale (ranging from “Frequent” to “Not Frequent”). Respondents must select one of three or four choices in items 14 and 15, respectively. An item example is, “Select the dot on the scale line below that, in your opinion, best represents the level of happiness in your current marriage”. The scale gradually moves from the center point, “happy,” which represents the level of happiness that most people feel in marriage, to the few who are extremely sad in marriage and the few who experience great joy or felicity in marriage. With an Cronbach alpha of 0.90, the instrument possesses excellent reliability.

The Relationship Satisfaction (RS) [81] scale is represented by 10 items. The Relationship Assessment Scale was designed to assess and measure an individual’s satisfaction with their relationship. It is a six-point scale where the one-dimensional range of “Strongly disagree” has been replaced by “Strongly agree”. For example, the item, “I am satisfied with the relationship to my partner” requires an evaluation of satisfaction; however, this satisfaction is based on the subject’s own values and criteria. With a Cronbach alpha of 0.94, the instrument is extremely reliable.

The Couple (Marital) Satisfaction Index is a 32-item scale that assesses a person’s satisfaction in the relationship with their partner [82], which is further referred to as their marital satisfaction. A six-point Likert scale has been used in this study (ranging from “1: always agree” to “6: always disagree”). An example of an item is, “How well does your partner meet your needs?”. With an Alpha Cronbach alpha of 0.93, the instrument offers excellent reliability. In this paper, we have opted to associate the variable assessed by this scale with marital satisfaction to avoid creating conceptual confusion between closely related psychological factors/variables.

The Family Distress Index (FDI) is a scale consisting of eight items that assess a family’s self-relationship with the onset of family difficulties (for instance, drug misuse, divorce, and emotional issues) and other challenges that reflect family disharmony and family intolerance; it was developed by [83]. The Family Distress Index (FDI) scale is used to obtain family challenges and problems and show distress or instability in the family. In this self-report scale, with a Likert scale ranging from one to four, participants stated how much their family has faced family-specific obstacles in the past year (from small problems to large problems). All of the variables are added together to obtain a score, with higher scores suggesting more family troubles. The items are arranged as follows: “Please select one of the following options to describe which of the family issues listed above has most recently affected your family in the past 12 months: 0, 1, 2, or 3”. An example of an item is: “Increasing the conversation between parents and children about how they disagree”. The instrument has a very good reliability with a Cronbach alpha of 0.93.

The Generalized Self-Efficacy Scale (SES), which was adapted to the role of the parent, describes a parent’s belief in the ability to successfully fulfill the parental role. The scale was devised in 1981 by Matthias Jerusalem and Ralf Schwarzer in German, and has since been utilized in various investigations, including having been modified for 33 other languages. The SES scale was considered to evaluate self-efficacy in adjusting to everyday issues, confidence in setting objectives, investing effort, and tenacity in actions. Participants responded to the 10 items on a four-point Likert scale, ranging from total disagreement to absolute agreement. Participants demonstrate a high level of self-efficacy if they receive a high score [84]. An item example is: “If I work hard enough and frequently enough, I always manage to solve difficult problems”. The SES has a strong reliability, with a Cronbach alpha of 0.92.

Mowen’s Personality Scale [85] was used to measure the respondents’ personalities according to the Big Five Model. This scale is made up of 15 elements that are used to assess the 5 dimensions of personality through 3 items. It is a Likert-type scale with 7 steps, 1 indicating total disagreement and 7 indicating entire agreement; the rating is performed by capitalizing the scores for each dimension. The instructions of the scale are as follows: “The descriptions speak to the individual’s qualities. You are kindly requested to carefully consider each characteristic and move in accordance with how well each of these descriptions fits the overall simulation or composition method you are using, assigning a number between 1 and 7”. An example of an item is: “Emotions fluctuate from extremely low to extremely high”. The instrument possesses a high level of reliability in all dimensions with a Cronbach alpha of 0.82 for the openness, 0.77 for the conscientiousness, 0.79 for the extraversion, 0.70 for the neuroticism, and 0.78 for the agreeableness dimensions.

### 2.3. Procedure

Participants were recruited through an online questionnaire. We targeted individuals who were over 18 years old, were married in the range between 5 and 10 years, and had at least one child classified as a minor living together with them. The invitation also provided a link to the study’s webpage, which described the study’s goals and the objectives of the questionnaires used. The participants provided informed consent, and the condition of confidentiality was met. Informed consent was gained by asking each individual if they were willing to participate in the study. If they agreed, they were permitted to proceed to the next level of the questionnaire; otherwise, they were obliged to exit the survey.

We have opted to employ convenience sampling to target possible participants who were available. This sampling strategy has the advantage of allowing researchers to swiftly discover and recruit participants. One disadvantage of the convenience sampling method is that the sample may not be representative of the total population, leading to skewed findings [65]. Because this study was conducted with internet-recruited people who freely participated, traditional sampling could still provide appropriate information in the sense that the major inclusion criterion was to have been married for 5–10 years. As a consequence, the information gathered was based on a self-selected sample of people who completed the survey. As extensions to the general linear model, a multiple linear regression and multivariate regression analyses were performed in this study. A high sample size is required for multiple regression to fully rule out chance as an explanatory mechanism in establishing the connection between predictors and the response variable [86]. In one study, a sample containing at least 92 participants was declared acceptable [87].

### 2.4. Analysis Plan

The collected data were inputted into the SPSS version 20 software (Chicago, IL, USA) for Windows for the analysis. For all of the variables that were measured, means and standard deviations were calculated. For the categorical variables, the frequencies and percentages were calculated [88].

Several predictive variables were present in the dataset used in the analysis. We utilized the multiple regression approach, which allowed the predictors’ cumulative influence on the dependent variable to be evaluated. Because the regression model included many variables, multivariate comparisons were possible, which possibly minimized the incidence of type I errors [89]. The goal of the data analysis was to identify whether or not there was a link between a group of independent variables or predictors and a single dependent variable; when the dependent variable was graded on a scale from one to ten, it is referred to as a continuous variable, and the independent variables are measured on a dichotomous, interval, or ratio scale; multiple regression is the suitable analysis for this. The conventional method of multiple regression analysis was used in this study, which involved the simultaneous addition of several predictors in the same model. We analyzed each independent in relation to the variation in the dependent variable using t-tests, which the other predictors could not account for [90]. The effect of each predictor was measured using beta coefficients.

## 3. Results

The means and standard deviations for each variable are shown in Table 1, along with the correlations between the study variables.

A three-step multiple linear regression model was created in order to respond to the first hypothesis. The correlation coefficients are presented in Table 1 and the regression coefficients are depicted in Table 2. Relationship satisfaction was predicted by three personality dimensions, namely agreeableness, openness, and conscientiousness (r^2^ = 0.28). When the attachment styles were added to the personality dimensions in the regression model in Model 2, the explanation in the variation of relationship satisfaction increased (r^2^ = 0.71), and when marital adjustment was added in Model 3 in the prediction model’s explanatory power, the overall model improved its predictiveness for relationship satisfaction (r^2^ = 0.78). This means that agreeableness, openness, conscientiousness (*F* = 31.71, *p* = 0.000), anxious and avoidant attachment (*F* = 121.83, *p* = 0.000), and negative marital adjustment (*F* = 142.36, *p* = 0.000) (as we have obtained in Table 3, there is a negative significant coefficient of r = −0.649 between marital satisfaction and marital adjustment) predict relationship satisfaction. Beta coefficients are also depicted in Table 3.

The results presented in Table 3 indicate that openness partially mediated the relationship between marital adjustment and relationship satisfaction (*R* = 0.65; *F* = 171.0; *p* = 0.00). An increased score on the openness dimension predicted the relationship satisfaction and a decreased marital adjustment, meaning that the more openness one of the partners shows, the more likely they were to avoid compromise in their relationship, thus a decreased score on marital adjustment.

A person with a high score on the openness dimension of personality is a person whose thoughts, feelings, interests, and actions are diverse; they have mental flexibility, the ability to quickly adapt to various contexts, and are a creative, imaginative, and nonconformist person. As a result, people who have a high level of openness are more adaptable in their relationships and have higher levels of relationship satisfaction.

According to the findings, openness partially mediated the association between marital adjustment and relationship satisfaction (*R* = 0.65; *F* = 171.0; *p* = 0.00), namely, the direct path between the independent variable and dependent variable remained significant even after the addition of openness as a mediator in the equation. An increased score on the openness dimension predicted both increased relationship satisfaction and decreased marital adjustment, although the direct path between the two types of satisfaction remained significant.

A three-step multiple linear regression model was created to address hypothesis three (Table 4). In Model 1, marital (couple) satisfaction variation was explained by the personality dimensions of agreeableness, openness, and conscientiousness (r^2^ = 0.21; *F* = 22.06; *p* = 0.00); anxious attachment and avoidant attachment were negative and significant predictors, and when added in Model 2, they contributed to an increased explanation in the variation of the marital satisfaction (r^2^ = 0.57; *F* = 63.93; *p* = 0.00). Lastly, in Model 3, when the marital adjustment score was added, the explanatory power increased (r^2^ = 0.62; *F* = 68.04; *p* = 0.00) and the whole model was predictive for the marital satisfaction. As the anxious and avoidant attachment increases, marital satisfaction decreases.

With the observation that both relationship satisfaction and marital satisfaction are both predicted by the same set of psychological predictors and having similar mechanisms, we further examined how parental self-efficacy was related to relationship satisfaction and marital satisfaction. Namely, we further investigated whether parental self-efficacy represented a mediator between relationship satisfaction and marital (couple) satisfaction.

Parental self-efficacy appeared to partially mediate the association between relationship satisfaction and marital satisfaction, according to the findings of this hypothesis (*R* = 0.88; *F* = 777.0; *p* = 0.00), with the results being depicted in Table 5. Our partial mediation result indicated that the direct path between the independent variable and dependent variable remained significant even after the addition of parental self-efficacy as a mediator in the equation. An increased parental self-efficacy score predicts a relationship satisfaction and increased marital satisfaction, although the direct path between the two types of satisfaction remained significant. The higher the parental self-efficacy, the higher the satisfaction in the relationship, which leads to an increase in satisfaction in the couple. Better self-efficacy is associated with a strong sense of personal effectiveness, greater achievement and better social integration, which all reflects on the well-being of the couple [91]. Parental self-efficacy is linked to a profound commitment to activities and an innate interest in the couple’s children. Married parents with a high feeling of self-efficacy stimulate the diversity of goals and maintain a strong commitment to them, supporting resiliency [91]. This characterized attitude of people with high self-efficacy generates personal achievements, reduces stress, and decreases vulnerability to depression, thus increasing relationship satisfaction. When the satisfaction in the relationship increases, the overall satisfaction in the couple will increase.

## 4. Discussion

The statistical data supported all five of the hypotheses proposed in this study.

This study was a non-experimental research study with a quantitative design. A benefit of using a non-experimental/survey design is that it requires only a one-time observation [92]. In this study, an experimental design would be useless, as establishing a cause-and-effect relationship would be nearly impossible. The lack of a control group is a disadvantage of the non-experimental design [92]. A qualitative approach was also ruled out because of the naturalistic observations or open-ended interviews, which would have yielded more meaningful answers and personal interpretations, and could have yielded a large number of responses that would have been difficult to classify and report in a coherent and concise manner [92].

The end of the twentieth century saw the emergence of the first models appeared that tried to explain how a married couple operates and the factors that ensure its stability. The models aimed at explaining the mechanisms underlying marital life (seen as a dynamic process over time) accounted for a number of antecedents of marital quality and stability, such as: the socio-demographic context in which partners enter into marital relationships, personal and interpersonal skills of partners, personality traits and behavior patterns, and the experiences they had in the home family environment [42,57,93].

Thus, an individual’s satisfaction with the relationships that they establish is higher in the case of people characterized by empathy, tolerance, interpersonal trust, and honesty, these being the peculiarities of an agreeable personality. For a relationship that involves ongoing role negotiations and where compromises are necessary for optimal functioning to be declared satisfactory, it is necessary for people to have the ability to understand the needs of the partner, to self-decentralize and embrace the other’s perspective; in other words, to be empathetic. Honesty is a facet of the agreeableness dimension that is the ingredient needed to achieve a relationship with a great amount of satisfaction. The feeling of being understood and valued, as well as the confidence that one can rely on their partner in difficult and less difficult times make relationship satisfaction higher. The responsibility, prudence, and reflexivity that are constituents of the conscientiousness dimension are also associated with the satisfaction in the relationship, as such people resort to a reflective processing of information when making decisions, including decisions regarding the relationship domain; they relate with prudence and have a good ability to analyze the factors that could lead the relationship into a difficult context, while also having good stress management skills. Furthermore, the more open the person is to new explanations, the wider the interests, and the greater the desire to develop and learn from different contexts, the greater the satisfaction in a relationship.

In conclusion with respect to personality factors, we present that the agreeableness (empathy, tolerance, honesty, and modesty), conscientiousness (responsibility and rationality in decisions), and openness (broad interests, creativity, and imagination) dimensions explain some of the variances of relational satisfaction. Aside from personality traits such as cognitive processes, affect, and behavior, the attachment styles contribute to predicting the relational satisfaction.

Openness partially mediates the relation among marital adjustment and relationship satisfaction. This result shows that the marital adjustment or adaptation is related to the relational satisfaction, and this relation is mediated by openness. In the case of people that are open to experiences, curious, and have broad and unconventional interests, they declare a high level of relational satisfaction. The ability to adapt to a relationship and to be open to new experiences guarantees an increased level of perceived relational satisfaction. Similarly, extraversion, another dimension of personality characterized by an increased level of energy that is oriented towards relationships and the social environment, is regarded as a mediator in relationships by the factors of sociability, good communication skills, and need for social recognition in the link between relational adaptability and satisfaction.

We also observed that marital satisfaction is predicted by the same three personality traits, attachment styles, and marital adjustment. Basically, the explanatory mechanism is similar to the one above, where the criterion variable was relationship satisfaction. Another important aspect of married life is the parental self-efficacy, which is the belief that partners can succeed and achieve good results in marital relationships. The predictive model for parental self-efficacy that this study highlighted consisted of individual factors, where personality traits and relationship satisfaction had a negative effect, and relationship satisfaction, anxious and avoidant attachment, and marital satisfaction had a positive effect. This predictive model for parental self-efficacy did not include marital adjustment, which has been demonstrated to be a weak predictor of parental self-efficacy. In conclusion, we can say that marital adjustment, a significant aspect in married life, along with individual factors and attachment, provides an explanation for relationship satisfaction and marital satisfaction. Moreover, it has been proven that parental self-efficacy can mediate the association between relationship satisfaction and marital satisfaction.

## 5. Conclusions

Several limitations are recognized in this study. First, a convenience sampling was conducted, where individuals had to independently agree to complete the questionnaires and participate this investigation.

An important limitation of this study is the convenience sampling used due to the circumstances associated with the COVID-19 pandemic as well as social distancing protocols. The sample consisted of married individuals (not dyads) who were also parents and who were available and willing to participate while the questionnaire was being distributed. In addition, the questionnaire was self-administered and independently completed by each participant according to their interpretations, thus some answers may show a tendency toward social desirability. However, to ensure the questionnaire was delivered to the largest segment of married individuals with children, it was distributed through public social media platforms.

Furthermore, the small sample size of the study weakens the statistical power of the calculated coefficients obtained in the findings, meaning that the findings cannot be applied to the entire Romanian adult population. The research has a cross-sectional design, the data being collected only once, which is a limitation, because there can be no inferences about causal links.

An important methodological limitation of our research is that our research design did not consider the spillover hypothesis: that the relationship between parents can affect only parents but not their relationship with their children (i.e., independent contexts), or that the relationship with parents can also affect children (i.e., related contexts).

Despite these limitations, the current study makes a significant contribution to couples therapies and physical and mental health interventions and provides insight into the experiences and perspectives of married individuals with children in Romania.

This quantitative study investigated parenting correlations, personality traits, self-efficacy, and relationship satisfaction, in addition to marital satisfaction. It also showed the predictors of a satisfactory marital relationship and the mediating factors of marital and relational satisfaction. This information is important in couples therapies and physical and mental health interventions. It has been commonly recognized that people who are happy in their relationships report decreased levels of stress, anxiety, and depression, as well as higher levels of life satisfaction and well-being [36,37]. Distress and tension in a relationship have been previously related to impaired immune system function and the development of later psychiatric illnesses in adulthood [94,95]. Furthermore, individuals who have experienced parental divorce as youths tend to be at a higher risk of doing the same in adulthood. Adults with divorced parents have problematic marriages, poor connections with their parents, lower levels of education, lower salaries, and report greater levels of psychological distress [96]. Some researchers believe that the decision of grandparents divorcing may have an effect on offspring two generations on. As a result, divorce is commonly linked to lower educational achievement, marital strife, and fewer parental relationships in future generations [96]. A number of studies have indicated that children flourish when they live with their birth or adoptive parents, as compared with children from other types of families [97,98]. Children of divorced parents tend to suffer from a number of issues, including scholastic challenges, aggressive behavior, depression, low self-esteem, stress, and poor social skills [99,100]. Parental disagreement also jeopardizes their child’s emotional stability, increasing the likelihood of social and psychiatric illnesses [88,101,102]. In general, when individuals are content with their interpersonal ties, the community benefits. Living partners have the ability to influence not only the moods of others, but also their behavioral and psychological well-being [37]. People who have satisfying relationships are less likely to become ill and have higher labor productivity. Increasing labor productivity, for the most part, leads to financial stability, which improves a person’s economic condition [103]. Those who are under financial stress are more likely to experience emotional distress [103]. People who are happy in their relationships are more likely to provide a healthy and stable environment for their children, which reduces the risk of abusive and harmful circumstances [104].

## Figures and Tables

**Table 1:** The descriptive statistics and correlations between variables.

Variables	*M*	*SD*	1	2	3	4	5	6	7	8	9	10	11	12
1. Anxious attachment	58.53	1.86	1											
2. Avoidant attachment	41.89	1.43	0.654 **											
3. Marital adjustment	37.47	0.39	0.386 **	0.497 **										
4. Relationship satisfaction	55.39	0.98	−0.663 **	−0.804 **	−0.649 **									
5. Parental self-efficacy	34.42	0.342	−0.387 **	−0.420 **	−0.365 **	0.495 **								
6. Marital satisfaction	126.07	1.81	−0.555 **	−0.732 **	−0.602 **	0.876 **	0.489 **							
7. Family distress	20.08	0.43	0.080	0.055	0.130 *	−0.132 *	−0.090	−0.137 *						
8. Openness	17.37	0.22	−0.309 **	−0.392 **	−0.246 **	0.499 **	0.496 **	0.416 **	−0.100					
9. Conscientiousness	17.27	0.23	−0.181 **	−0.288 **	−0.300 **	0.369 **	0.469 **	0.357 **	−0.085	0.383 **				
10. Introversion	12.87	0.33	0.196 **	0.158 *	0.071	−0.163 *	−0.125	−0.183 **	0.022	−0.175 **	0.085			
11. Neuroticism	11.34	0.32	0.295 **	0.218 **	0.188 **	−0.190 **	−0.114	−0.166 *	0.059	−0.053	0.007	0.305 **		
12. Agreeableness	17.82	0.20	−0.164 *	−0.250 **	−0.193 **	0.211 **	0.279 **	0.188 **	0.001	0.229 **	0.305 **	0.223 **	0.028	

**. At the 0.01 level, there is a correlation (2-tailed). *. At the 0.05 level, there is a correlation (2-tailed).

**Table 2:** The beta coefficients for the multiple linear regression analysis for the investigated variables: relationship satisfaction, openness, conscientiousness, agreeableness, anxious attachment, avoidant attachment, and marital adjustment.

Variable	B	95% CI	Beta	t	*p*
**Model 1**					
Openness	1.766	0.258, 0.412	0.412	6.848	0.000
Conscientiousness	0.819	0.261, 0.193	0.193	3.143	0.002
Agreeableness	0.274	0.280, 0.057	0.057	0.979	0.329
**Model 2**					
Openness	0.749	0.170, 0.175	0.175	4.410	0.000
Conscientiousness	0.469	0.164, 0.111	0.111	2.851	0.005
Agreeableness	−0.201	0.177, −0.042	−0.042	−1.139	0.256
Anxious attachment	−0.119	0.024, −0.226	−0.226	−4.954	0.000
Avoidant attachment	−0.388	0.033, −0.566	−0.566	−11.762	0.000
**Model 3**					
Openness	0.755	0.150, 0.176	0.176	5.049	0.000
Conscientiousness	0.262	0.147, 0.062	0.062	1.783	0.076
Agreeableness	−0.248	0.156, −0.052	−0.052	−1.594	0.112
Anxious attachment	−0.102	0.021, −0.194	−0.194	−4.805	0.000
Avoidant attachment	−0.313	0.030, −0.456	−0.456	−10.264	0.000
Marital adjustment	−0.740	0.090, −0.296	−0.296	−8.265	0.000

**Table 3:** A model of mediation for the investigated variables: personality, marital adjustment, and relationship satisfaction.

Path	r^2^	*F*	df	*p*	B	SE(B)	β	*p*	95% CI
**c**	0.42	171.9345	(1, 236)	<0.01	−1.6	0.12	−0.64	<0.01	−1.8, −1.3
**a**	0.06	15.1392	(1, 236)	<0.01	−0.14	0.03	−0.24	<0.01	−0.21, −0.07
**b & c′**	0.54	140.407	(2, 235)	<0.01					
**c′**					−1.4	0.11	−0.56	<0.01	−1.62, −1.17
**b**					1.5	0.19	0.36	<0.01	1.16, 1.93
**a** × **b**							−0.08		

r^2^ = explained variation/total variation; *F* = ANOVA; B = unstandardized coefficients; (SE) = standard error; β = standardized coefficients; (df) = degree of freedom; *p* = level of significance; 95% confidence interval (CI) = 95.0% confidence interval for B.

**Table 4:** The beta coefficients for the multiple linear regression analysis of the investigated variables: marital (couple) satisfaction, openness, conscientiousness, agreeableness, anxious attachment, avoidant attachment, marital adjustment.

Variable	B	95% CI	Beta	t	*p*
**Model 1**		0.258, 0.412			
Openness	2.537	2.537, 0.499	0.320	5.086	0.000
Conscientiousness	1.717	1.717, 0.504	0.219	3.407	0.001
Agreeableness	0.425	0.425, 0.541	0.048	0.785	0.433
**Model 2**					
Openness	0.842	0.842, 0.387	0.106	2.172	0.031
Conscientiousness	1.097	1.097, 0.375	0.140	2.926	0.004
Agreeableness	−0.392	−0.392, 0.403	−0.044	−0.972	0.332
Anxious attachment	−0.121	−0.121, 0.055	−0.125	−2.212	0.028
Avoidant attachment	−0.734	−0.734, 0.075	−0.580	−9.758	0.000
**Model 3**					
Openness	0.852	0.852, 0.360	0.108	2.367	0.019
Conscientiousness	0.727	0.727, 0.353	0.093	2.056	0.041
Agreeableness	−0.476	−0.476, 0.375	−0.054	−1.270	0.205
Anxious attachment	−0.091	−0.091, 0.051	−0.094	−1.777	0.077
Avoidant attachment	−0.599	−0.599, 0.073	−0.473	−8.174	0.000
Marital adjustment	−1.326	−1.326, 0.215	−0.287	−6.151	0.000

**Table 5:** The mediation model for the investigated variables: parental self-efficacy, relationship satisfaction, marital (couple) satisfaction.

Path	r^2^	*F*	Df	*p*	B	SE(B)	β	*p*	95% CI
**c**	0.76	777.58	(1, 236)	<0.01	1.6	0.05	0.87	<0.01	1.50, 1.73
**a**	0.24	76.54	(1, 236)	<0.01	0.17	0.01	0.49	<0.01	0.13, 0.21
**b & c′**	0.77	396.22	(2, 235)	<0.01					
**c′**					1.55	0.06	0.83	<0.01	1.4, 1.6
**b**					0.39	0.19	0.07	<0.01	0.01, 0.76
**a** × **b**							0.03		

## Data Availability

Not applicable.
